# Primary pulmonary myxoid sarcoma with *EWSR1-CREB1* fusion: a case report and review of the literature

**DOI:** 10.1186/s13000-020-00930-2

**Published:** 2020-02-10

**Authors:** Zhenwei Chen, Yihui Yang, Rongming Chen, Chi Sing Ng, Hongqi Shi

**Affiliations:** 1 Department of Pathology, Jinhua Municipal Central Hospital, 351 Mingyue Road, Jinhua, 321000 Zhejiang Province People’s Republic of China; 2 Department of Pathology, the People’s Hospital of Changfeng County, Changfeng County, Anhui Province People’s Republic of China; 3 Department of Pathology, St. Teresa’s Hospital, Kowloon, Hong Kong

**Keywords:** Pulmonary, Myxoid, Sarcoma, Chondrocyte-like, Physaliferous-like, *EWSR1*, *CREB1*

## Abstract

**Background:**

Primary pulmonary myxoid sarcoma (PPMS) is an extremely rare lung sarcoma that is characterized in most cases by recurrent balanced chromosomal translocation t(2;22)(q33;q12) leading to the oncogenic fusion gene *EWSR1-CREB1.*

**Case presentation:**

We report a case of PPMS with molecular confirmation using fluorescence in situ hybridization (FISH) and DNA sequencing in a 45-year-old female patient. Computer tomography (CT) scanning revealed a peripheral circumscribed solid mass of 2.1 × 2 cm in the right lung superior lobe. Histologically, the tumor cells ranged from stellate, polygonal to chondrocyte-like or physaliferous-like, forming reticular network of delicate lace-like cellular strands and cords in abundant myxoid stroma. The tumor cell immunophenotype was positive for vimentin, EMA and negative for CK-pan, TTF-1, CAM5.2, S-100, calponin, SMA, desmin, ALK, CD31 and CD34. Molecular analysis demonstrated *EWSR1-CREB1* gene fusion in this tumor. During 38 months of follow-up, the patient was alive with no clinical or radiological evidence of recurrence or metastasis.

**Conclusion:**

PPMS is a rare low-grade sarcoma with distinct histological and genetic features. We add another case to the literature of this rare tumor and report for the first time occurrence of chondrocyte-like and physaliferous-like tumor cells in this tumor, thus enriching its morphologic and cytologic spectrum.

## Background

Primary pulmonary sarcomas are extremely rare with prevalence of about 0.2% [1]. They comprise a heterogeneous group of sarcomas morphologically similar to the soft tissue counterparts. Among these rare sarcomas, primary pulmonary myxoid sarcoma (PPMS) are even more infrequent. PPMS is a recently described lung sarcoma more prevalent in young females with a characteristic genetic *EWSR1-CREB1* fusion in most cases [2, 3].. To the best of our knowledge, 25 cases have been reported in the English literature [2–11], among which only 17 cases were confirmed with presence of the *EWSR1-CREB1* gene fusion. We report another case of PPMS harboring the *EWSR1-CREB1* gene fusion confirmed by molecular method, with review of the literature.

## Case presentation

**Fig. 1:**
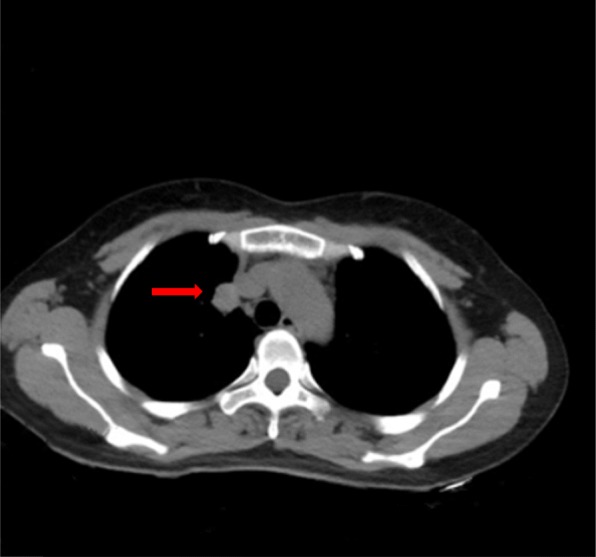
Chest computed tomography showed a 2.1 × 1.7 cm well-defined round mass exhibiting mild, heterogeneous internal enhancement at the periphery of the right superior lobe

A 45-year-old woman was referred to our hospital for physical checkup. She was asymptomatic with no pulmonary obstructive symptoms or pneumonia. The patient was a non-user of alcohol and tobacco products. General physical and laboratory examination was unremarkable. Chest computed tomography (CT) revealed a 2.1 × 2 cm peripheral solid mass in the right lung superior lobe featuring moderate heterogeneous enhancement (Fig. 1). There was no abnormality on bronchoscopic and cytological examinations. Routine blood tests and tumor marker levels were normal. Although CT findings suggested the possibility of hemangioma, the patient insisted on surgical excision. During surgery, a mass was detected in the right lung superior lobe which adhered to the superior vena cava with no invasion to the adjacent lung parenchyma or bronchus. Adjuvant chemotherapy was not given post-operatively. At follow-up 38 months after surgery, there was no evidence of recurrence or metastasis.

**Fig. 2:**
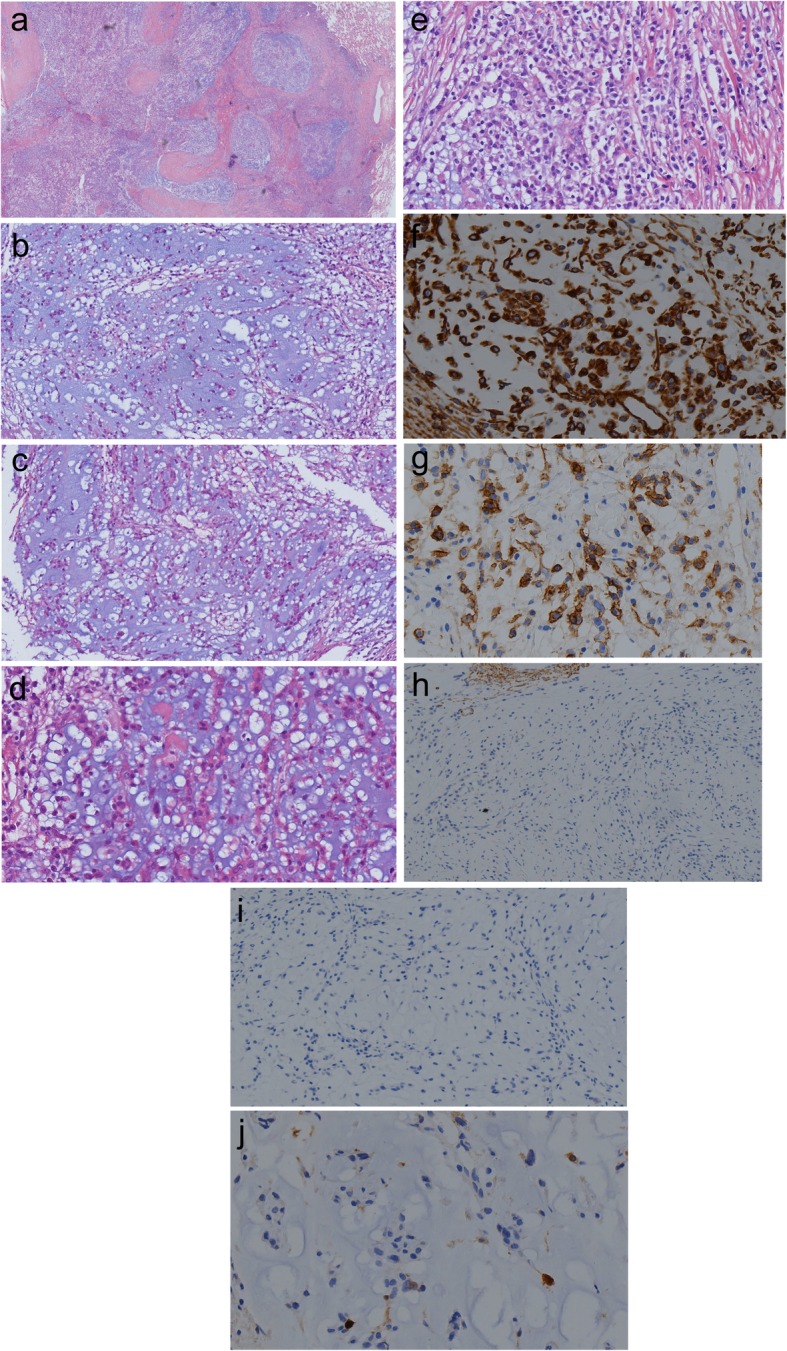
**a.** The tumor had abundant myxoid stroma and a multinodular architecture at low-magnification (**a**: magnification × 20 and **b**: magnification ×100). **b** & **c** & **d** The tumor showed variable cellularity with polygonal, stellate to chondrocyte-like or physaliferous-like tumor cells organized in prominent reticular network of delicate lace-like cellular strands and cords within prominent myxoid stroma (**b** & **c** magnification × 100, **d**: magnification × 400). **e.** Abundant lymphoid cells and plasma cells at the periphery or within the tumor (magnification × 400) **f.** Tumor cell immunohistochemical positive expression of vimentin (magnification × 400). **g.** Tumor cell immunohistochemical positive expression of EMA (magnification × 400). **h**. Tumor cell immunohistochemical negative expression of Desmin (magnification × 200). **i**. Tumor cell immunohistochemical negative expression of ALK (magnification × 400). **j**. Tumor cell immunohistochemical negative expression of S-100 (magnification × 400)

**Fig. 3:**
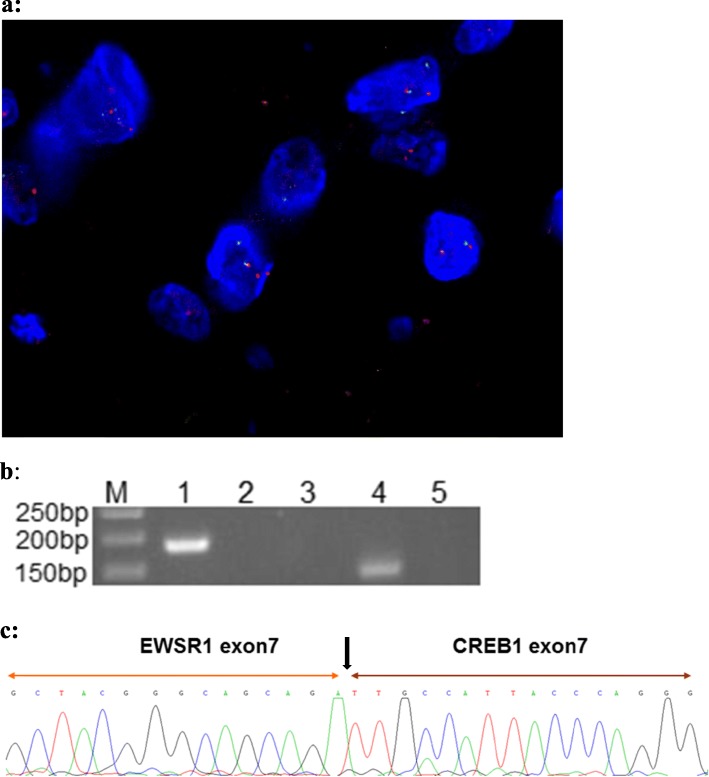
**a**: Dual color interphase fluorescence in situ hybridization utilizing the *EWSR1* break-apart probe. Split red and green signals within a single tumor cell demonstrated the presence of *EWSR1* rearrangement. **b**: Gel electrophoresis of the RT-PCR products using *EWSR1* and *CREB1* primers; confirming presence of *ESWR1-CREB1* fusion in the patient’s sample (Lane 4). M:50 bp markers; Lane 1: Internal control, PGK; Lane 2: Negative control with *EWSR1* exon 7 + *CREB1* exon 7 fusion primer; Lane 3: Negative control with *EWSR1* exon 7 + *CREB1* exon 8 fusion primer; Lane 4: Patient’s sample with *EWSR1* exon 7 + *CREB1* exon 7 fusion primer; Lane 5: Patient’s sample with *EWSR1* exon 7 + *CREB1* exon 8 fusion primer. **c:** Sanger sequencing result of the patient’s RT-PCR product demonstrated in Lane 4 of **b**. The sequence was the same as the *EWSR1* - *CREB1* fusion gene as reported in the literature

Macroscopically, the tumor was a solitary, well-circumscribed mass with a fleshy homogeneous white and gelatinous cut surface, measuring 2 × 1.7 × 1 cm without invasion to the bronchus. The tumor was histologically multinodular and composed of oval, or polygonal cells with vesicular nuclei, reminiscent of chondrocyte-like or physaliferous-like cells, in a background of myxoid stroma (Fig. 2a-d). There were rare mitotic figures and rich lymphoplasmacytic cell infiltration was also evident (Fig. 2e). There was no evidence an endobronchial component. Immunophenotypically, the tumor was positive for vimentin (Fig. 2f), epithelial membrane antigen (EMA) (Fig. 2g) and negative for CK-pan, CAM5.2, CD31, CD34, smooth muscle actin (SMA), desmin (Fig. 2h), anaplastic lymphoma kinase (ALK) (Fig. 2i), calponin, TTF-1 and S-100 protein (Fig. 2j) (Table 1). Molecular analysis was performed on formalin fixed paraffin embedded (FFPE) material by fluorescence in situ hybridization (FISH) using LSI *EWSR1* dual-color break-apart probe (Break Apart Rearrangement probe, Ambipin,China), and specific gene fusion transcripts by reverse transcription-polymerase chain reaction (RT-PCR). Primers for the RT-PCR were located in exon 7 of *EWSR1* (5’TCCTACAGCCAAGCTCCAAGTC3’) and in exon 7 of *CREB1* (5’GTACCCCATCGGTACCATTGT3’). FISH showed a clear separation of red and green signals within a single tumor cell, demonstrating the presence of a *EWSR1* gene rearrangement (Fig. 3a). The RT-PCR gene fusion products were confirmed by DNA sequencing with Sanger method (ABI3730, Japan) to show in-frame fusion of the 5′ region of *EWSR1* (exon 7) to the 3′ region of *CREB1* (exon 7) (Fig. 3b & c). The histological, immunophenotypic and molecular findings confirmed the diagnosis of primary pulmonary myxoid sarcoma with *EWSR1-CREB1* gene fusion.

**Table 1:** List of antibodies

Antigen	Clone	Dilution	Manufacturer
Vimentin	MX034	1:200	Fuzhou MXB Biotechnology Co,Ltd. China
EMA	GP1.4	1:100	Fuzhou MXB Biotechnology Co,Ltd. China
CK-pan	polyclonal	1:200	Fuzhou MXB Biotechnology Co,Ltd. China
CAM5.2	CAM5.2	1:100	Fuzhou MXB Biotechnology Co,Ltd. China
CD31	JC/70A	1:100	Fuzhou MXB Biotechnology Co,Ltd. China
CD34	QBEnd/10	1:100	Fuzhou MXB Biotechnology Co,Ltd. China
SMA	1A4	1:200	Fuzhou MXB Biotechnology Co,Ltd. China
Calponin	MX023	1:300	Fuzhou MXB Biotechnology Co,Ltd. China
TTF-1	8G7G3/1	1:200	Fuzhou MXB Biotechnology Co,Ltd. China
S-100	polyclonal	1:200	Fuzhou MXB Biotechnology Co,Ltd. China
Desmin	D33	1:200	Guangzhou Onco Care Biotechnology Co,Ltd. China
ALK	MX064	1:200	Guangzhou Onco Care Biotechnology Co,Ltd. China

## Discussion

Nicholson et al. first described a rare primary pulmonary myxoid sarcoma in 1999 as a novel low-grade malignant myxoid endobronchial neoplasm [4]. Further studies showed that this tumor has a characteristic genetic fingerprint characterized by the oncogenic fusion gene *EWSR1-CREB1* [2], which has been first included in the latest WHO fascicle as a characteristic of this tumor [12]. To date, 25 cases of this sarcoma have been described in the English literature [2–11],with only 17 confirmed by presence of *EWSR1-CREB1* fusion [2, 3, 5–10]. Our case is the eighteenth case of PPMS with confirmed *EWSR1-CREB1* gene fusion. All 26 cases (including the 5 cases with no *EWSR1-CREB1* gene fusion, 1 case with *ESWR1* rearrangement not involving *CREB1* and 2 cases without molecular genetic workup) are reviewed and summarized in Table 2. The patients had a broad age range from 21 to 80 years (mean 43 years), with female predominance (female: male, 1.5:1). The clinical manifestations are relatively nonspecific and include cough, hemoptysis and weight loss. There was no definite site of predilection of this tumor in the lung. Although initially reported mostly as endobronchial growths related to the bronchial tree, there were some tumors not associated with the bronchus [7, 8, 10], as in our case. One such case was a *EWSR1-CREB1* gene rearranged low grade myxoid sarcoma of the pulmonary artery [10]. Grossly, the excised lesions are well circumscribed, ranged in size from 1.5 to 14 cm (average 4 cm) with pale, glistening cut surface. Microscopically, the characteristic features of most cases are lobulated architecture with cords of ovoid, spindle or stellate cells embedded in a prominent myxoid matrix. Most cases have a patchy background of inflammatory cells consisting mainly of lymphocytes and plasma cells. In distinct contrast, the tumor cells in our case are chondrocyte-like or physaliferous-like. Predominance of chondrocyte-like and physaliferous-like tumor cells in PPMS has not been reported in the literature previously. The inflammatory infiltrate in our case is also more intense. These unique findings of our case enrich the morphologic and cytologic spectrum of PPMS. Regarding genetics of PPMS, genetic study was not performed in two of the 26 cases. Among the remaining 24 cases, 18 (including our case) showed *EWSR1-CREB1* gene fusion, one showed *EWSR1* rearrangement but not fusion with *CREB1* (case 16) [6], and 5 were negative for *ESWR1-CREB1* gene fusion. This makes a 75% positive rate for *EWSR1-CREB1* fusion, 79% for *EWSR1* rearrangement, and a negative rate of 21% for *ESWR1-CREB1* fusion or ESWR1 rearrangement in the 24 cases where genetic study was performed in the reviewed series of PPMS. These findings are broadly similar to the previously reported *EWSR1-CREB1* gene fusion rate of 70% [2] and 63% [3], and *EWSR1* and/or *CREB1* gene rearrangement rate of 89% [3] in two smaller series of PPMS. Thway K et al [2] detected *EWSR1-CREB1* fusion gene in 7 PPMS, with the fusion loci located in exon 7 of EWSR1 and exon 7 of CREB1 in 6 of the 7 cases. One other case showed fusion loci in exon 7 of EWSR1 and exon 8 of CREB1. There was no histological difference in the tumors bearing the different gene fusions. Furthermore, one case in this series with no *EWSR1-CREB1* fusion died from brain metastasis, while another case bearing the *EWSR1-CREB1* fusion of the same series had kidney metastasis but was alive and well after 3 years [2]. In another series [7], one case with *EWSR1-CREB1* fusion developed metastasis to the contralateral lung, but remained disease free 72 months after removal of the lung metastasis. It appears that PPMS with *EWSR1-CREB1* fusion fares better than those without *EWSR1-CREB1* fusion. However, whether presence of *EWSR1-CREB1* fusion connotates better prognosis in PPMS requires further study.

**Table 2:** Clinicopathological and genetic features of current case and previously reported cases of primary pulmonary myxoid sarcoma

Case No. (Ref. No.)	Age	sex	EBC (+/−)	Size (cm)	Pathological features	IHC	Molecular genetics	Treatment	Follow-up (months)
1(3)	27	F	EBC+	4	Ill-defined nodules of myxoid stroma, interweaving cords of small uniform, round or slightly elongated cells with eosinophilic cytoplasm, occasional mitoses.	Vimentin+, CK-, S100-, desmin-, SMA-, CD34-.	ND	Surgery	NEOD after 36
2(3)	43	F	EBC+	13	Ill-defined nodules of myxoid stroma, interweaving cords of small uniform, round or slightly elongated cells with eosinophilic cytoplasm, occasional mitoses.	Vimentin+, CK-, S100-, desmin-, SMA-, CD34-.	ND	Surgery	NEOD after 6
3(2)	27	F	EBC+	4	Well circumscribed, lobulated, reticular network with delicate lacelike strands and cords of cells within prominent myxoid stroma, tumor cells showed no or minimal atypia.	CK-, S100-, desmin-.	EWSR1-CREB1 fusion	Surgery	NEOD after 180
4(2)	33	F	EBC+	3.5	Lobulated, reticular network with delicate lacelike strands and cords of cells within prominent myxoid stroma, tumor cells showed mild to moderate atypia.	CK-, S100-, desmin-.	EWSR1-CREB1 fusion	Surgery	NEOD after 144
5(2)	45	F	EBC+	1.5	Circumscribed, lobulated, cellular sheets or patternless, tumor cells showed mild to moderate atypia.	S100 focal+, CK-, desmin-, p63-.	Negative	Surgery	NEOD after 12
6(2)	36	F	NR	NR	Circumscribed with fibrous pseudocapsule. Reticular network with delicate lacelike strands and cords of cells within prominent myxoid stroma, tumor cells showed minimal atypia.	CK-, EMA-, TTF1-, S100-, desmin-.	Negative	Surgery	DOD with brain metastases a few months after diagnosis
7(2)	32	F	EBC+	NR	Lobulated. Reticular network with delicate lacelike strands and cords of cells within prominent myxoid stroma, tumor cells showed moderate atypia.	CK-, EMA-,S100-, desmin-.	EWSR1-CREB1 fusion	Surgery	NR
8(2)	28	M	EBC+	2.8	Infiltrative and lobulated, cellular sheets or patternless, tumor cells showed mild to moderate atypia,	EMA weak+, CK-, TTF1-, S100-, HMB45-, melan A-, desmin-.	EWSR1-CREB1 fusion	Surgery	Left renal metastasis, alive and well after 3 years.
9(2)	67	M	EBC+	2.8	Well circumscribed and lobulated, reticular network with delicate lacelike strands and cords of cells within a prominent myxoid stroma., tumor cells showed minimal atypia.	EMA weak+, CK-, TTF-1-, S100-, desmin-.	EWSR1-CREB1 fusion	Surgery	NR
10(2)	68	F	EBC+	2.0	Well circumscribed and lobulated, reticular network with delicate lacelike strands and cords of cells within a prominent myxoid stroma, tumor cells showed moderate to marked atypia.	EMA weak+, CK-, p63-, TTF-1-, S100-, desmin-.	Negative	Surgery	NR
11(2)	63	F	EBC+	NR	Lobulated, cellular sheets or patternless, tunor cells showed mild to minimal atypia.	EMA weak +, CK-, TTF-1-, S100-, HMB45-, melan A-, desmin-.	EWSR1-CREB1 fusion	Surgery	NEOD after 48
12(2)	51	M	NR	2.0	Well circumscribed and lobulated, reticular network with delicate lacelike strands and cords of cells within a prominent myxoid stroma, tumor cells showed mild to moderate atypia.	NR	EWSR1-CREB1 fusion	Surgery	NR
13(5)	31	M	EBC+	2.7	Well circumscribed, reticular cords of oval, short spindle or polygonal cells with mild atypia, rare mitotic figures, an abundant myxoid stroma, scattered lymphoplasmacytic infiltrates.	Vimentin+, EMA focal+, CK-, TTF-1-, napsin A-, S-100-, CD34-, desmin-, SMA-, CD10-, p63-, calponin- caldesmon-, c-kit-, HMB-45-, synaptophysin-, GFAP-	EWSR1-CREB1 fusion	Surgery	NEOD after 68
14(6)	66	F	EBC+	4	Polygonal to spindled cells, reticular network with delicate lacelike strands and cords of cells within a prominent myxoid stroma, tumor cells showed mild atypia.	EMA focal+, CK-, p63-, S100-, desmin-.	EWSR1-CREB1 fusion	Surgery	NR
15(6)	28	M	NR	8.5	Lobulated, biphasic, ~ 40% composed of myxoid pools, exuberant fibroinflammatory reaction with confluent plasma cells, tumor cells showed moderate atypia.	Desmin+, EMA focal+, CK-, p63-. S100-.	Negative	Surgery	NR
16(6)	28	M	EBC+	6	Infiltrative, focal necrosis and inflammation, tumor cells showed severe atypia.	EMA focal+, CK-, p63-, S100-.	EWSR1 rearrangement,. but not CREB1	Surgery	NR
17(7)	26	M	EBC+	9	Multinodular, reticular network with delicate lacelike strands and cords of cells within prominent myxoid stroma, tumor cells showed mild to moderate atypia.	Vimentin+, EMA focal+, CD99 focal weak+, SMA-, desmin-, caldesmon-H-, calponin-, S100-, CK-, CD31-, CD34-, p63-, CD56-, synaptophysin-.	EWSR1-CREB1 fusion	Surgery	NEOD after 19
18(7)	49	F	EBC-	4	Multinodular, reticular network with delicate lacelike strands and cords of cells within prominent myxoid stroma, tumor cells showed mild to moderate atypia.	Vimentin+, EMA focal+, CD99 focal weak+, SMA focal+, desmin-, caldesmon-H, calponin-, S100-, CK-, CD31-, CD34-, p63-, CD56-, synaptophysin-.	EWSR1-CREB1 fusion	Surgery	NEOD after 117
19(7)	54	F	EBC+	4.5	Multinodular, reticular network with delicate lacelike strands and cords of cells within prominent myxoid stroma, tumor cells showed moderate atypia.	Vimentin+, EMA focal+, CD99 focal+, SMA-, desmin-, caldesmon-H-, calponin-, S100-, CK-, CD31-, CD34-, p63-, CD56-, synaptophysin-.	EWSR1-CREB1 fusion	Surgery	NEOD after 152
20(7)	65	M	EBC+	13	Multinodular, reticular network with delicate lacelike strands and cords of cells within prominent myxoid stroma, tumor cells showed mild atypia.	Vimentin+, EMA focal+, CD99 focal+, SMA-, desmin-, caldesmon-H-, calponin-, S100-, CK-, CD31-, CD34-, p63-, CD56-, synaptophysin-.	EWSR1-CREB1 fusion	Surgery	Metastasis to contralateral lung, NEOD 72 months after removal of metastasis.
21(8)	29	F	EBC-	3	Well circumscribed, short spindle, ovoid or stellate cells in reticular network, myxoid stroma, lymphoplasmacytic infiltration, tumor cells showed mild atypia.	Vimentin+, EMA+. SMA-, SMMHC-, calretinin-, TTF-1-, CK-, p63-, S-100-, CD34-, CD56-.	EWSR1-CREB1 fusion	Surgery	NEOD after 17
22(4)	80	F	EBC+	NR	Multinodular, spindle cells arranged in reticular pattern within prominent myxoid stroma, tumor cells showed moderately atypia	Vimentin+, EMA focal+. CK-, S100-, HMB45-, CD31-, CD34-, SMA-, caldesmon-H-, desmin-, GFAP-.	EWSR1-CREB1 fusion	Surgery	NEOD after 36.
23(9)	32	F	NR	3.5	Well-delineated lobulated, anastomosing cords and small nests of epithelioid cells admixed with stellate cells in chondromyxoid matrix.	Vimentin+, CD68 weak+, CD163 weak+, synaptophysin weak+. CK-, EMA-, calponin-, GFAP-, SMA-, desmin-, caldesmon-H-, S-100-, HMB-45-, CD34-, CD31-, chromogranin-.	EWSR1-CREB1 fusion	Surgery	NEOD after 96.
24(10)	21	F	PA/EBC-	NR	Polypoid tumor, trabecular networks, rare solid areas, tumor cells showed oval nuclei and eosinophilic cytoplasm,	CK weak+, SMA+, INI1-, EMA-, S100-, desmin-, ERG-, MDM2-, CDK4-.	EWSR1-CREB1 fusion	Surgery	NEOD after 38
25(11)	48	M	NR	14	Bland looking medium-sized oval to round epithelioid cells arranged in prominent reticular and microcystic lace-like chordoid pattern in highly myxoid stroma.	Vimentin+, CD10 focal+、EMA focal+, CK-, TTF-1-, ERG-, CD31-, p63-, desmin-, SMA-, S100-, CD34-, CD30- , MUC4-, TLE1-, STAT6-.	Negative	Surgery	NEOD after 23
26 (our case)	45	F	EBC-	2.1	Well circumscribed, multinodular, reticular network of delicate lace-like cellular strands and cords in abundant myxoid stroma, chondrocyte or physaliferous-like tumor cells with mild atypia.	Vimentin+, EMA+, CK-, TTF-1-, CAM5.2-, S-100-, calponin-, SMA-, desmin-, ALK-, CD31-, CD34-.	EWSR1-CREB1 fusion	Surgery	NEOD after 38

*F* female, *M* male, *EBC*+/− endobronchial component involved (+) or not involved (−). *IHC* immunohistochemical stains, *ND* not done, *NR* not reported, *PA* pulmonary artery, *DOD* died of disease, *NEOD* no evidence of disease, *CK* cytokeratin(s), *EMA* epithelial membrane antigen, *SMA* smooth muscle actin, *SMMHC* smooth muscle myosin heavy chain, *TTF-1* thyroid transcription factor-1

PPMS should be differentiated from extraskeletal myxoid chondrosarcoma (EMC), which can also arise in the lung [13, 14]. Histologically, EMC is composed of cords of cells with scarce cytoplasm immersed in abundant myxoid matrix similar to PPMS. EMC, however, at least focally expresses S-100 which is negative in PPMS, as in our case. Genetically, EMC may harbor *EWSR1-NR4A3、TAF15-NR4A3 or TFG-NR4A3* gene fusion [15, 16], and may thus confound with PPMS. However, PPMS exhibits the characteristic *EWSR1-CREB1* fusion gene, allowing distinction from EMC. Differentiation between PPMS and angiomatoid fibrous histiocytoma (AFH), which may occur in the lung [17], is more problematic. Although both tumors are located predominantly endobronchially and harbor the *EWSR1-CREB1* fusion gene, they differ in morphology. PPMS is composed of cords and clusters of spindle, stellate, ovoid, and in our case chondrocyte-like and physaliferous-like tumor cells arranged in a reticular pattern within prominent alcian blue positive myxoid stroma. In contrast, AFH comprises sheets and islands of spindle to epithelioid cells with bland ovoid vesicular nuclei and abundant eosinophilic cytoplasm within loose stroma. PPMS has more abundant myxoid stroma and lacks the prominent peripheral cuff of lymphocytes usually present in AFH. Moreover, desmin is expressed in 50% of AFH, which is not present in PPMS. Furthermore, ALK expression is common in AFH, which is negative in PPMS. Finally, AFH may show other fusion genes, such as *EWSR1-ATF1* and *FUS-ATF1*, both of which are absent in PPMS. PPMS should be also be distinguished from myoepithelial tumors, which can also arise in the lung [18] with endobronchial growth pattern and *EWSR1* rearrangements. Myoepithelial tumors are immunohistochemically positive for CK, p63, SMA, calponin and S-100 protein, which are negative in PPMS. Pulmonary microcystic fibromyxoma (PMF) are composed of bland spindle to stellate cells with uniform nuclei widely spaced within fibromyxoid stroma which is alcian blue positive and hyaluronidase sensitive [19]. However, PMF are not endobronchially located and are much less cellular with bland stellate cells disposed in a microcystic pattern. Furthermore, PMF does not harbor the *EWSR1-CREB1* gene fusion, distinct from PPMS. Another differential diagnosis is inflammatory myofibroblastic tumor (IMT) which may possess myxoid stroma and inflammatory infiltrates. The stellate tumor cells in IMT are immunophenotypically positive for SMA, desmin and ALK, which are negative in PPMS. Since the physaliferous-like tumor cells in PPMS exhibit cytoplasmic bubbles, they can potentially be confused with lipoblasts. PPMS thus needs to be differentiated from low grade primary or metastatic myxoid liposarcoma [20]. Myxoid liposarcoma, however, often harbors rearrangement involving the *DDIT3* gene, which is not present in PPMS. The various differential diagnoses and their features are summarized in Table 3.

**Table 3:** Salient features of primary pulmonary myxoid sarcoma and differential diagnoses

Tumors	Pathological features	IHC	Molecular genetics	Differentiating features from PPMS
Primary pulmonary myxoid sarcoma (PPMS)	Well-circumscribed, lobulated, reticular network of delicate lace-like cellular strands and cords in abundant myxoid stroma, tumor cells are stellate, polygonal with also chondrocyte-like or physaliferous-like tumor cells first reported in our case, prominent lymphoplasmacytic infiltrates within and at periphery of tumor.	Vimentin+, EMA+, CK--, TTF-1-, S-100-, calponin-, SMA-, desmin-, ALK-, CD31-, CD34-.	*EWSR1-CREB1* fusion	NA
Extraskeletal myxoid chondrosarcoma (EMC)	Well-circumscribed, multinodular, tumor lobules separated by fibrous septae, umor cells epithelioid to spindled arranged in cords, strands, or clusters embedded in abundant myxoid stroma.	Vimentin+, S-100+, rarely EMA+, keratins+.	*,EWSR1-NR4A3,* *TFG-NR4A3,* *HSPA8-NR4A3, TCF12-NR4A3,* *FUS-NR4A3 or TAF15-NR4A3* gene fusion	S-100+, different molecular genetics. Rare as primary in lungs, may present as lung metastasis.
Angiomatoid fibrous histiocytoma (AFH)	Sheets and islands of spindle to epithelioid cells with bland ovoid vesicular nuclei and abundant eosinophilic cytoplasm within loose myxoid stroma.	ALK+, desmin+.	*EWSR1-ATF1, FUS-ATF1* gene fusion	No lobular or reticular architecture, no chondrocyte-like or physaliferous-like cells, ALK+ and desmin+, with different molecular genetics in AFH.
Myoepithelial tumors (MT)	Well-circumscribed, solid sheets, nested or cord-like growth pattern, hyalinized or myxoid stroma, moderate to severe nuclear pleomorphism.	Cytokeratins+, EMA+, S100+, calponin+, SMA+, p63+, GFAP+.	*EWSR1-FUS,* *EWSR1-PBX1, EWSR1-ZNF444, EWSR1-POU5F1* gene fusions	No reticular pattern, no chondrocyte-like or physaliferous-like cells, different 1HC and molecular genetics in MT.
Pulmonary microcystic fibromyxoma (PMF)	Well-circumscribed, bland spindled to stellate cells widely spaced within prominent fibromyxoid stroma with prominent cystic change.	Vimentin+, CD34-, CD31-, HMB45-, SMA-, desmin-, S-100-, ALK-, CKpan-, EMA-, calretinin-, TTF1-	none	Prominent cystic pattern, much less cellular, no chondrocyte-like or physaliferous-like cells, no diagnostic molecular genetic change and not endobronchially located in PMF.
Inflammatory myofibroblastic tumor (IMT)	Areas of myxoid stroma with prominent vessels or hyalinized collagenous stroma, and contain a prominent infltrate of plasma cells and lymphocytes.	SMA+, desmin+, ALK+, rarely keratins+	*RANBP2-ALK, RRBP1-ALK,* *ETV6-NTRK3* gene fusions	No reticular pattern, prominent inflammatory component, no chondrocyte-like or physaliferous-like cells, SMA+, ALK+ with different molecular genetics in IMT.
Low grade myxoid liposarcoma (LGML)	Large well-circumscribed, monotonous small ovoid cells with fine chromatin, inconspicuous nucleoli, and scant cytoplasm., many characteristic lipoblasts., prominent plexiform vasculature, myxoid background with areas of mucin pooling, imparting a “pulmonary edema-like” pattern.	S-100+, rarely MDM2+ and CDK4+	*DDIT3*-*FUS* and *DDIT3*-*EWSR1* gene fusions	Characteristic prominent plexiform vasculature, “pulmonary edema-like” pattern, S-100+, different molecular genetics in LGML. Lung is rare site for primary LGML.

*NA* not applicable, *CK* cytokeratin, *EMA* epithelial membrane antigen, *SMA* smooth muscle actin

Although most patients treated by adequate surgery had no local recurrences, three patients developed metastasis within a follow up period from 4 months to 15 years. The metastatic sites included the contralateral lung [7], the left kidney and brain [2]. The patient with brain metastasis died a few months after surgery. The patient with metastasis to the left kidney is still alive and well [2]. The patient with metastasis to the contralateral lung occurred 7 months after surgery [7]. Though PPMS is considered a low-grade sarcoma, it appears that there are no reliable histological or clinical features for predicting its prognosis and outcome. Some cases histologically showing nuclear atypia, necrosis and high mitotic figures, with increased Ki-67 proliferative index did not fare worse prognosis [2]. On the other hand, endobronchial location, capsule-like fibrosis, absence of solid architecture and lack of necrosis might be predictive of more favorable outcome, though presence of endobronchial component and capsule-like features had also been observed in metastatic cases [2]. Molecular fingerprint may play a prognostic role, as patients with *EWSR1* rearrangement [11] or *EWSR1-CREB1* gene fusion [2, 7] may have more favorable prognosis, while those with no *EWSR1-CREB1* fusion or wild type *EWSR1* may portend poor clinical outcome [2, 11].

## Conclusion

PPMS is a rare low-grade sarcoma that occurs mostly in middle age women with most harboring the characteristic *EWSR1-CREB1* fusion gene. Our case adds to the literature of this rare tumor and enriches its morphologic and cytologic spectrum with occurrence of chondrocyte-like and physaliferous-like tumor cells. Early diagnosis of PPMS requires high index of suspicion in pulmonary myxoid tumors and is based on clinical features, histological and molecular studies. The clinical behavior of this tumor is still uncertain due to its rarity and limited follow up of reported cases. There are no reliable histological or clinical features to predict prognosis and outcome of this tumor.

## Data Availability

The dataset supporting the conclusion of this article is included within the article.

## Abbreviations

AFH: Angiomatoid fibrous histiocytoma
ALK: Anaplastic lymphoma kinase
CAM5.2: Cytokeratin
CK-pan: Cytokeratin (Pan)
CT: Computer tomography
EMA: Epithelial membrane antigen
EMC: Extraskeletal myxoid chondrosarcoma
FFPE: Formalin fixed and paraffin embedded
FISH: Fluorescence in situ hybridization
IMT: Inflammatory myofibroblastic tumor
PMF: Pulmonary microcystic fibromyxoma
PPMS: Primary pulmonary myxoid sarcoma
RT-PCR: Reverse transcription-polymerase chain reaction
SMA: Smooth muscle actin
TTF-1: Thyroid transcription factor 1
